# Preparation and Characterization of Highly Elastic Foams with Enhanced Electromagnetic Wave Absorption Based On Ethylene-Propylene-Diene-Monomer Rubber Filled with Barium Titanate/Multiwall Carbon Nanotube Hybrid

**DOI:** 10.3390/polym12102278

**Published:** 2020-10-03

**Authors:** Hasti Bizhani, Ali Asghar Katbab, Emil Lopez-Hernandez, Jose Miguel Miranda, Miguel A. Lopez-Manchado, Raquel Verdejo

**Affiliations:** 1 Department of Polymer Engineering and Color Technology, Amirkabir University of Technology, Tehran 1591634311, Iran; bijani@aut.ac.ir; 2 Institute of Polymer Science and Technology (ICTP-CSIC), C/Juan de la Cierva 3, 28006 Madrid, Spain; emil.lopez@ictp.csic.es (E.L.-H.); lmanchado@ictp.csic.es (M.A.L.-M.); 3 Department Estructura de la Materia, Facultad de Fisicas, Universidad Complutense de Madrid, 28040 Madrid, Spain; miranda@ucm.es

**Keywords:** EPDM, barium titanate, multiwall carbon nanotubes, foams, electromagnetic interference shielding

## Abstract

Hybrid ethylene-propylene-diene-monomer (EPDM) nanocomposite foams were produced via compression molding with enhanced electromagnetic wave absorption efficiency. The hybrid filler, consisting of 20 phr ferroelectric barium titanate (BT) and various loading fractions of multi-wall carbon nanotubes (MWCNTs), synergistically increased the electromagnetic (EM) wave absorption characteristics of the EPDM foam. Accordingly, while the EPDM foam filled with 20 phr BT was transparent to the EM wave within the frequency range of 8.2–12.4 GHz (X-band), the hybrid EPDM nanocomposite foam loaded with 20 phr BT and 10 phr MWCNTs presented a total shielding effectiveness (SE) of ~22.3 dB compared to ~16.0 dB of the MWCNTs (10 phr). This synergistic effect is suggested to be due to the segregation of MWCNT networks within the cellular structure of EPDM, resulting in enhanced electrical conductivity, and also high dielectric permittivity of the foam imparted by the BT particles. Moreover, the total SE of the BT/MWCNTs loaded foam samples remained almost unchanged when subjected to repeated bending due to the elastic recovery behavior of the crosslinked EPDM foamed nanocomposites.

## 1. Introduction

In recent years, the high influx of electronic devices has increased the number of electromagnetic (EM) contaminants around the world, which is called electromagnetic interference (EMI) [1]. EMI can disturb the performance of electronic devices, and stop them from functioning. Metals, which have traditionally been used to counteract the negative effects of EM waves, have disadvantages such as high cost and weight, low corrosion resistance, and most importantly, their inability to absorb electromagnetic waves [2]. Thus, conductive polymer nanocomposites have been studied as potential EM absorbers due to their lightness, high corrosion and chemical resistance, ease of processability, and low production cost. Additionally, these materials are able to absorb and dissipate EM wave energy to heat [3,4]. 

Several papers have discussed the effect of foaming upon EM wave absorption and shielding characteristics of polymer nanocomposites and reported higher EMI shielding effectiveness (SE) compared to their solid counterparts within the X-band frequency region (8.2–12.4 GHz), which is widely applicable in various industries such as aerospace and communications. These results have been ascribed to the high specific surface area of porous structures toward the incident EM wave and the occurrence of multiple-reflections of the wave within the material [5]. Current trends towards further improvement of EMI shielding are directed to the use of hybrid fillers, comprising of conductive/conductive or ceramic/conductive fillers [6,7,8,9,10,11,12,13,14,15,16]. Although these studies have reported synergistic effect (“1 + 1 > 2” phenomenon) for these types of hybrid filler systems in EMI shielding, a low number of works have analyzed polymer nanocomposites with cellular structure [7,13,16,17].

Barium titanate (BT) is a ferroelectric ceramic, with high dielectric constant, relatively low dielectric loss, and excellent polarization characteristics; hence, it is widely employed in thermistors, capacitors, and transducers [18,19]. However, polymer matrices filled with high electrical conductive CNTs exhibit high power loss behavior (tanδ) as a result of the leakage current passing through the nanocomposite [20]. Therefore, incorporating a combination of BT with CNTs into the polymer materials is expected to promote both conductive and dielectric properties of the polymers and, hence, enhance the EM wave absorbing behavior. Meanwhile, EPDM rubber is known as an olefinic elastomer with remarkable ozone/water and light resistance due to a very low unsaturated structure. Hence, its usage as a matrix should enable the fabrication of a highly deformable and elastic EMI wave absorber with low water absorption [21]. Moreover, this elastomer is able to be chemically crosslinked during the foaming by a peroxide curing system. To the best of our knowledge, no work has been reported on the fabrication and characterization of polymer foams filled with a hybrid of BT and MWCNTs as an EM wave absorber within the high-frequency region. Therefore, in the present work, medium unsaturated EPDM rubber was selected as the hosting polymer matrix and was filled separately with BT, MWCNTs, and the hybrid of these two fillers for the preparation of foamed samples. The dielectric properties and EM wave absorption characteristics of the EPDM foams comprising BT/MWCNTs hybrid were analyzed over the X-band frequency region. Moreover, the effect of repeated mechanical bending upon the stability of foam structure as well as EM wave absorption behavior was studied. 

## 2. Experimental Section/Methods

*Materials*: Commercial EPDM rubber, Keltan 2750, with 48 wt.% ethylene content, 7.8 wt.% ethylidene-norbornene (ENB) as diene monomer and mooney viscosity (ML, 1 + 4, 125 °C) of 28, density of 0.86 g/cm^3^, was kindly supplied by Lanxess (Geleen, Netherlands). Maleic anhydride grafted EPDM (MA–g–EPDM), Royaltuf^®^ 498, with 0.8–1.2 wt.% maleic anhydride content, mooney viscosity of 30 (ML 1 + 4, 125 °C), and density of 0.87 g/cm^3^, was purchased from Crompton—Uniroyal Chemical Co. (Middlebury, CT, USA). 4,4′-Oxybis(benzenesulfonyl hydrazide) (OBSH) (GENITRON^®^ OB), also provided by Lanxess (Leverkusen, Germany), was used as an inorganic blowing agent. BT powder with a mean particle diameter of less than 3 μm was purchased from Sigma-Aldrich (product number 208108, St. Louis, MO, USA). Finally, MWCNTs, Nanocyl 7000, with 90% purity, 9.5 nm average diameter, 1.5 μm average length, and 250–300 m^2^ surface area, were purchased from Nanocyl SA, Sambreville, Belgium.

*Formulations and samples preparation*: Compounding of EPDM with BT/MWCNTs, MWCNTs, and BT was carried out using a laboratory two-roll mill at 70 °C, according to the method presented in ASTM D3182. First, MA–g-EPDM was mixed with previously heated and masticated EPDM rubber. Then, dried BT and MWCNTs were incorporated into the rubber band, followed by the addition of the curing ingredients and blowing agent (Table 1). All milled compounds were left for 24 h before foaming. The simultaneous foaming and curing processes were carried out in a hydraulic press at 160 °C and 200 bar, using the previously measured optimum cure time (t_90_) for each compound.

*Characterization*: The crystal structure of BT was characterized by Raman spectroscopy using a Renishaw InVia Confocal Raman microscope (Renishaw plc, Wotton-under-Edge, UK). The analyses were performed at 514 nm excitation wavelength and resolution of 0.02 cm^−1^.

The porous structure of the foamed BT/MWCNTs, BT, and MWCNTs nanocomposites was examined using a scanning electron microscope (Philips ESEM XL30, Amsterdam, Netherlands) at 25 kV. The prepared foamed samples were cryo-fractured in liquid nitrogen, and the fractured surfaces were Au/Pd sputter-coated. The images were processed using Digimizer software to analyze the cells diameter and distribution as well as cells density. The cell density was calculated by using the following relationship:

N = (nM^2^/A)^1.5^ (1)

where n, M, and A are denoted as the number of cells, magnification factor, and area, respectively.

AC electrical conductivity of the cured BT/MWCNTs, BT, and MWCNTs nanocomposite foams was measured using an ALPHA high-resolution Novocontrol broadband dielectric spectrometer (Novocontrol Technologies GmbH, Hundsangen, Germany) at room temperature over a frequency range region of 10^−1^ Hz–10 MHz. Cylindrical samples, 22 ± 0.1 mm in diameter and 1 ± 0.1 mm thickness, were placed in the dielectric cell between two parallel gold-plated electrodes, 20 and 30 mm in diameter for the top and bottom electrodes, respectively, and applying 1 V as the amplitude of the AC electrical current. To eliminate contamination, the electrodes and specimen surfaces were cleaned with ethanol before testing.

The EMI SE properties were obtained by measuring the real and imaginary parts of the dielectric permittivity (ɛ′ and ɛ″) and magnetic permeability (μ′ and μ″) within the X-band frequency range employing a vector network analyzer (Agilent E8364B). The test was conducted on 3 mm thick samples and the results are the average of five samples for each foam. The measurement errors were, on average, less than 3%, for all the samples over the X-band frequency region. To investigate the effect of repeated bending on EMI SE, the hybrid nanocomposite foam, containing 10 phr MWCNTs, was subjected to 1000 bending with a radius of 2.0 mm and the EMI SE was obtained.

The thermal conductivity of samples was measured in accordance with ASTM E 1530 using FOX 50 thermal conductivity analyzer on cylindrical samples, 51 ± 0.1 mm diameter and 12.5 ± 0.1 mm thickness. The experimental error of this measurement is less than 5% of the absolute value.

## 3. Results and Discussion

**Figure 1:**
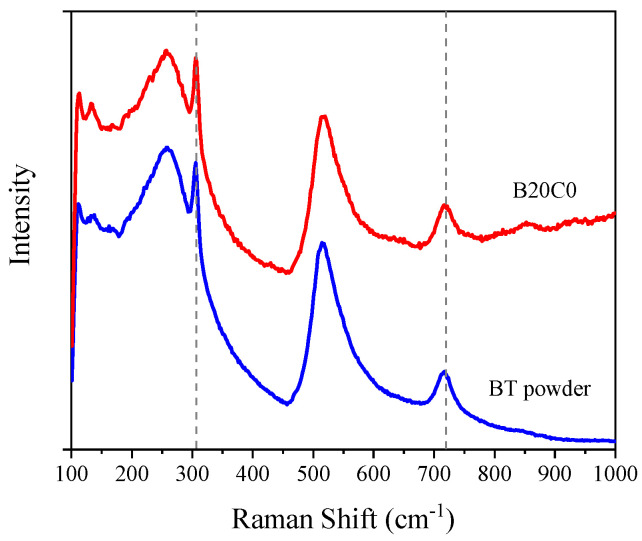
Raman spectra of barium titanate (BT) powder and cellular B20C0 composite.

Among the different crystal structures of BT, the tetragonal type presents higher polarization ability and microwave absorbing properties when subjected to an external EM wave [22,23]. As the Curie temperature of BT (120–130 °C) is relatively low, we first examined the possible effects of the compounding process upon its crystalline structure. For this purpose, raman spectroscopy, which has been known as a powerful tool to analyze the crystalline structure of BT [24], was performed on the neat BT powder and corresponding BT filled EPDM foam. Figure 1 shows the spectra of BT powder and EPDM foam filled with 20 phr BT (B20C0) with no significant difference between the absorption peaks at 305 and 718 cm^−1^, which are assigned to the tetragonal phase of BT [25]. This evidences that the compounding and foaming processes have not affected the tetragonal structure of BT crystals and, hence, its piezoelectric behavior is expected to remain unchanged. 

**Figure 2:**
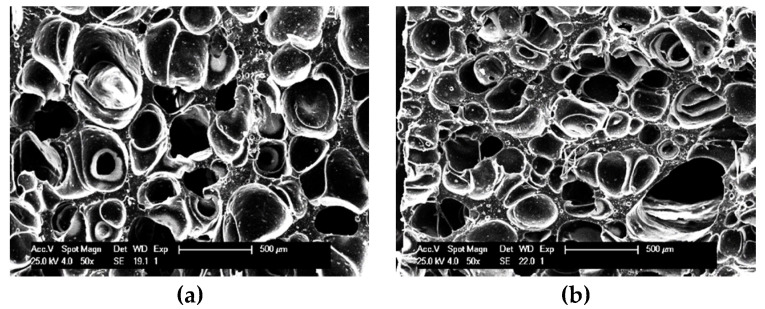

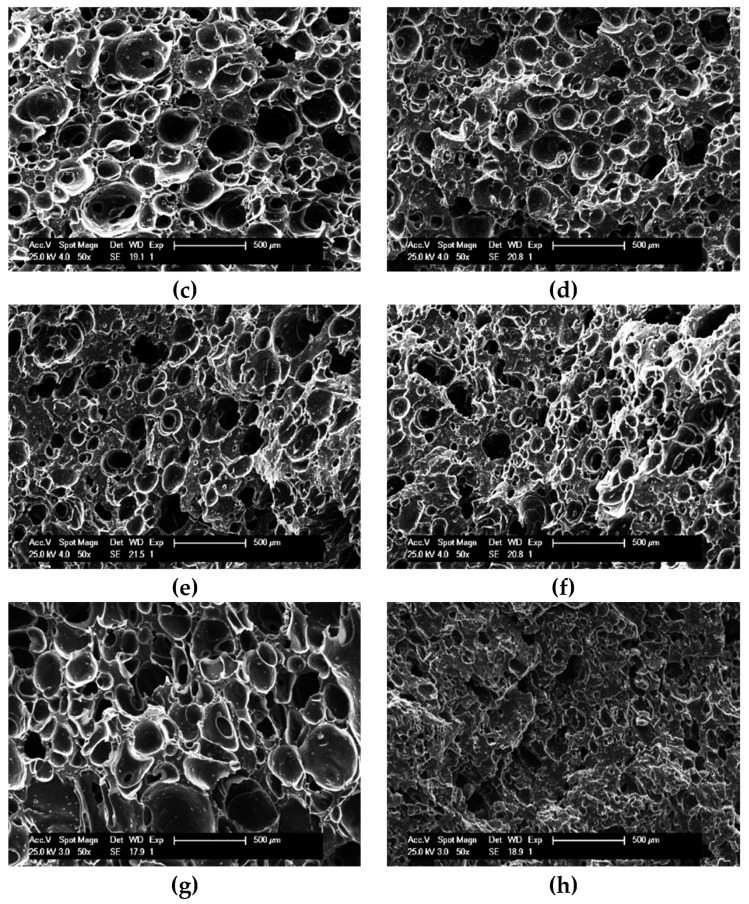
SEM micrographs of cryo-fractured surfaced foamed nanocomposites: (**a**) B20C0, (**b**) B20C2, (**c**) B20C4, (**d**) B20C6, (**e**) B20C8, (**f**) B20C10, (**g**) B0C2, and (**h**) B0C10.

**Figure 3:**
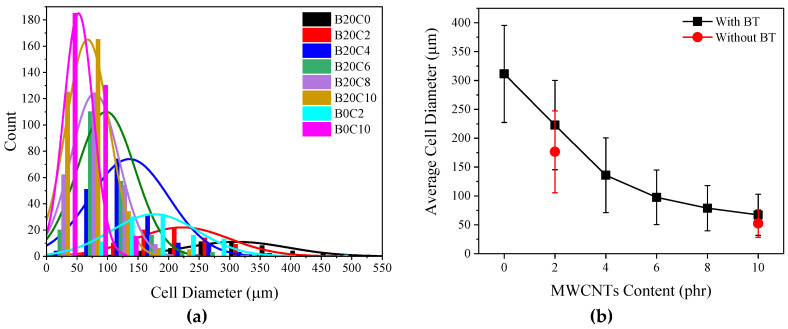

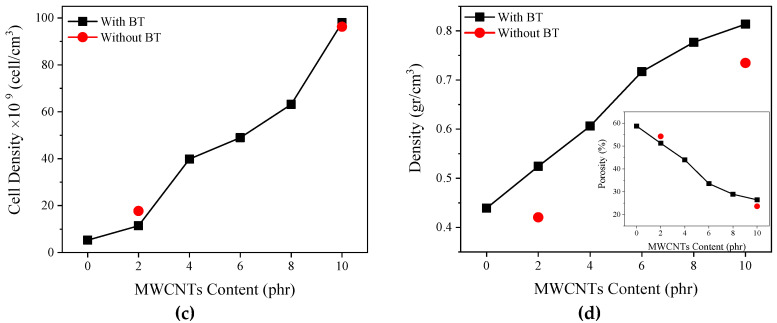
(**a**) Cells size distribution, (**b**) average cell size, (**c**) cell density, and (**d**) density; of ethylene-propylene-diene-monomer (EPDM)/multi-wall carbon nanotubes (MWCNT)/BT and EPDM/MWCNT foams containing different MWCNT loadings.

Figure 2 compares the SEM micrographs of the EPDM foamed samples comprising different amounts of BT/MWCNTs hybrid. The images were further analyzed to determine the foam morphology including cell size, distribution, and cell density (Figure 3). As can be observed, the cells sizes decrease from 311 ± 84 μm to 67 ± 35μm for the foamed samples containing 20 phr BT and 20 phr BT/10 phr MWCNTs, respectively. Moreover, as observed in Figure 3a, the presence of MWCNTs resulted in smaller cell sizes and a narrower cell size distribution, implying a more homogeneous cell morphology. These results together with the increase in the cells density (Figure 3c) are indicative of the nucleating effect of the CNT particles during the foaming process [26]. Similar results have also been reported for foam nanocomposites composed of various types of nanofillers fabricated using both chemical and physical (supercritical carbon dioxide) blowing agents [26,27,28]. The influence of the MWCNTs on the density and porosity of the foams is also presented in Figure 3d, where an opposite trend is observed. All these results, morphology and density, are ascribed to the reduction in curing scorch time, and hence the onset of the crosslinking reaction by the presence of MWCNTs particles, which limits the time for the foam expansion within the mold. This has been reported and discussed in our recently published paper [29]. Meanwhile, the apparent higher density of the samples composed of BT is due to the higher density of the BT (6.02 gr/cm^3^). Moreover, these results lead to the conclusion that BT does not play any significant role in the development of the cellular structure of the EPDM foams compared with MWCNTs. 

**Figure 4:**
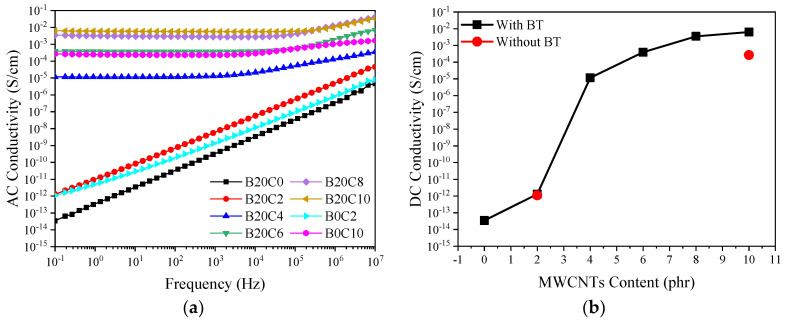
(**a**) AC conductivity (σac) vs. frequency for BT/MWCNTs and MWCNT nanocomposite foams in the frequency region of 10^−1^ to 10^7^ Hz; (**b**) DC electrical conductivity of BT/MWCNTs and MWCNTs nanocomposite foams as a function of MWCNTs content.

Electrical conductivity is a vital index to evaluate the potential EMI absorption and shielding performance of conductive nanocomposites. The AC conductivity (σ_AC_) of a dielectric material presents the extent of its frequency-dependent behavior. Figure 4a displays the variation of AC conductivity as a function of frequency for the prepared foamed samples. As can be observed, above 2 phr MWCNTs, the foams exhibit frequency-independent behavior (insulation-conduction transition) implying the formation of interconnected conductive paths [30]. This behavior is commonly described in terms of the percolation of CNT particles throughout the polymer matrix [6]. Additionally, the DC electrical conductivity (σ_DC_) of the samples can be extracted by the following relationship

σ_AC_ = σ_DC_ + Aω^s^ (2)

where ω = 2πf, f is frequency, A is a constant, and s is a power-law exponent (s = 1 for polymers). Thus, σ_DC_ value can be obtained by extrapolating the σ_AC_ to low frequencies (Figure 4b). The insulating nature of samples below the percolation threshold of the MWCNTs (≤2 phr) is clearly observed with DC conductivities below 10^−12^ S/cm. As the percolation threshold is passed, the DC electrical conductivity increases several orders of magnitude to 10^−5^ S/cm, 4 × 10^−4^ S/cm, and 9 × 10^−3^ for the foamed samples filled with 4 phr, 10 phr MWCNTs and 20 phr BT/10 phr MWCNTs, respectively, which are suitable for EM wave absorbing applications [31,32]. Therefore, the DC conductivity of the hybrid sample containing 10 phr of MWCNTs is one order of magnitude higher than the sample without BT. Furthermore, the sample based on B20C6 hybrid system shows similar AC values to that of the sample composed of only 10 phr MWCNTs (B0C10). These results imply the synergistic effect of BT and MWCNTs upon the conductivity of the foams. This could be ascribed to a dual excluded volume effect, originated from the formation of foam microstructure as well as the presence of the micro-size BT particles. In other words, the evolution of the cellular structure and the presence of the BT particles restricts the available volume for the MWCNTs and segregates them towards the cell walls and edges [5,33]. The segregation of conductive fillers during the foaming process has previously been observed as a factor to reduce the percolation threshold compared to their counterpart solid samples [5,16,29,34], and has also been recently reported for polypropylene (PP) foams containing hybrids of carbon black (CB) and MWCNTs [35]. Moreover, considering the excluded volume theory [36,37], the presence of a secondary particulate filler, such as BT, would further reduce the available volume for the CNT particles. Similar results have been reported for PP/BT/MWCNTs solid nanocomposites [38] and PP/MWCNTs/CB foam samples [35]. In these studies, this type of behavior has been ascribed to the improved electrical conductivity as a result of enhanced connectivity between the one-dimensional MWCNTs in conjunction with the zero-dimensional particles. This improved connectivity has been modeled based on a Voronoi geometry, resulting from Swiss cheese model, combined with the percolation theory [39]. Based on this model the improved conductivity for the hybrid filler systems depends on the secondary filler size and was favored with micro-size particulate fillers, such as the BT particles used in the present work. 

**Figure 5:**
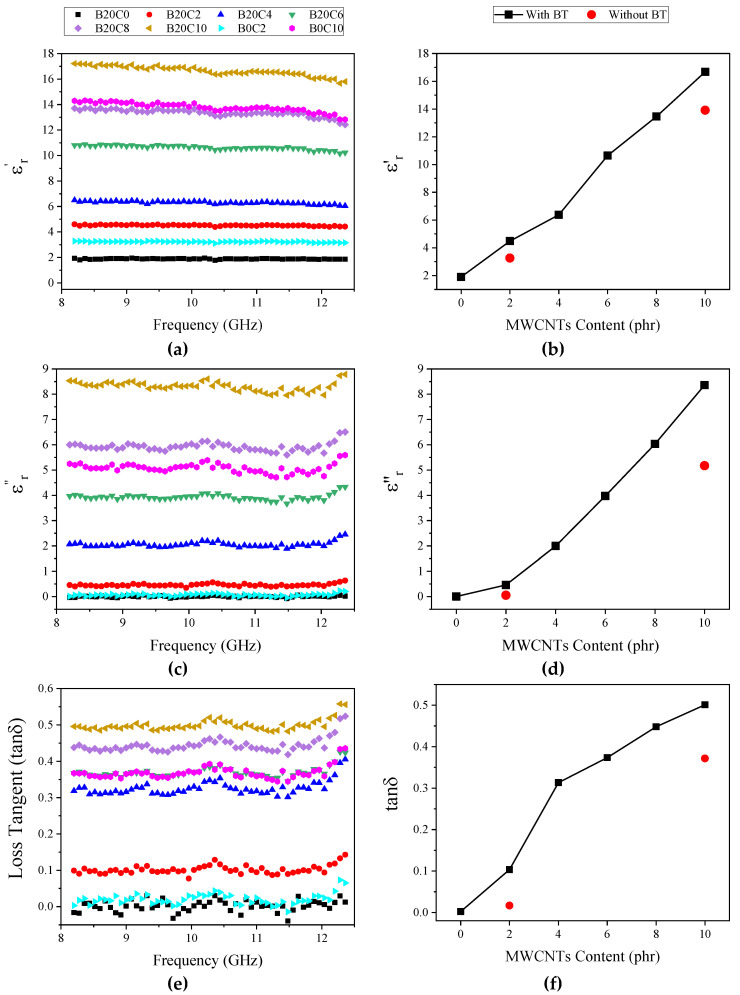
Dielectric permittivity of the foamed samples in the X-band: (**a**,**b**) real part, (**c**,**d**) imaginary part, (**e**,**f**) loss tangent as a function of the frequency (**a**,**c**,**e**) and as a function of the MWCNTs loading fraction at 10 GHz (**b**,**d**,**f**).

The material ability to save and dissipate EM energy is related to both its dielectric permittivity and magnetic permeability. Therefore, both parameters were measured to analyze the EMI shielding efficiency (SE) for our prepared foamed samples. The real and imaginary parts of the relative permeability were found to be 1 and 0, respectively, within the X-band frequencies, due to the absence of magnetic filler. Figure 5 demonstrates the variation of both real (ε_r_′) and imaginary (ε_r_″) components of the dielectric permittivity over the X-band. In our recently published paper [29], the values of ε_r_′ and ε_r_″ for the unfilled EPDM foam showed to be 1.6 and 0.001, respectively, which were slightly lower than the B20C0 foam (ε_r_′ = 1.9 and ε_r_″ = 0.0045). Meanwhile, the incorporation of MWCNTs into the composition of the EPDM/BT foams led to an increase in ε_r_′, ε_r_″, and dielectric loss factor (tan delta). Such increases are indicative of an enhanced degree of polarization, which is ascribed to two simultaneous polarization processes: a rotational polarization, from the dipoles attached to the surface of BT and MWCNTs, and interfacial space charge polarization, from the interface between the two fillers. The increase in ε_r_′ is indicative of the higher extent of polarization of the samples due to the presence of both ferroelectric BT particles and MWCNTs. In other words, both MWCNTs and BT particles can behave as carriers of polarizable dipoles within the polymer matrix, whereas the increase in the ε_r_″ would be the result of both polarization [15], and conductance loss [40]. Therefore, the increase in permittivity components (ε_r_′ and ε_r_″) is also reflected in the loss tangent (tan δ = ε_r_″/ε_r_′) (Figure 5e), which is a reasonable criterion of the intrinsic ability of a material to dissipate EM energy. Figure 5b,d,f represent the variation of the dielectric parameters (ε_r_′, ε_r_″, and tan δ), with MWCNTs content at 10 GHz, respectively. As can be observed, the nanocomposite originated from the BT/MWCNTs hybrid exhibits higher values for e′, e″, and tan delta than counterpart samples composed of only MWCNTs, which evidences the synergistic effect of these two fillers on dielectric properties of the foamed EPDM composites.

The EMI shielding effectiveness is the result of three mechanisms, including reflection of the incident wave from the surface of the shield as a result of the impedance mismatch between the air and the material, and absorption in which the energy of EM wave is dissipated and transformed into thermal energy while passing through the shield. The third is the multiple reflections of the waves at various surfaces or interfaces within the shield, i.e., air cells and nanoparticles. Therefore, the total SE (SET) can be expressed as follows [41,42]:

SE_T_ = SE_A_ + SE_R_ + SE_M_ (3)

where SE_A_, SE_R_, and SE_M_ are the shielding effectivenesses by absorption, reflection, and multiple reflections, respectively, which are defined as [42]:

SE_T_ = 10log(P_i_/P_t_) (4)

SE_A_ = 20loge^t/^^σ^ (5)

SE_R_ = 39.5 + 10log(σ/2fπμ) (6)

**Figure 6:**
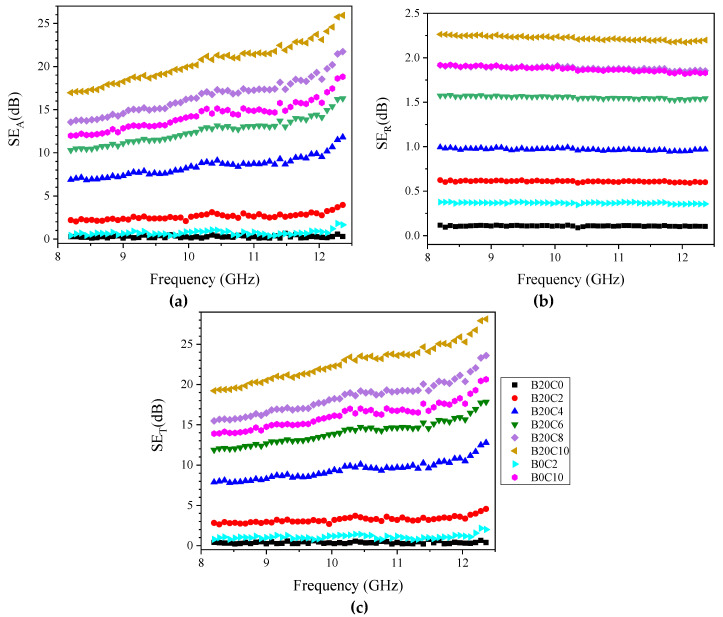
(**a**) SE_A_, (**b**) SE_R_ and (**c**) SE_T_ of the developed foams with 9 mm thickness within the X-band frequency.

**Figure 7:**
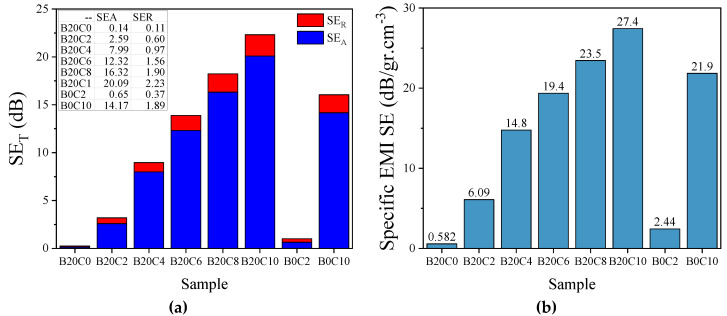

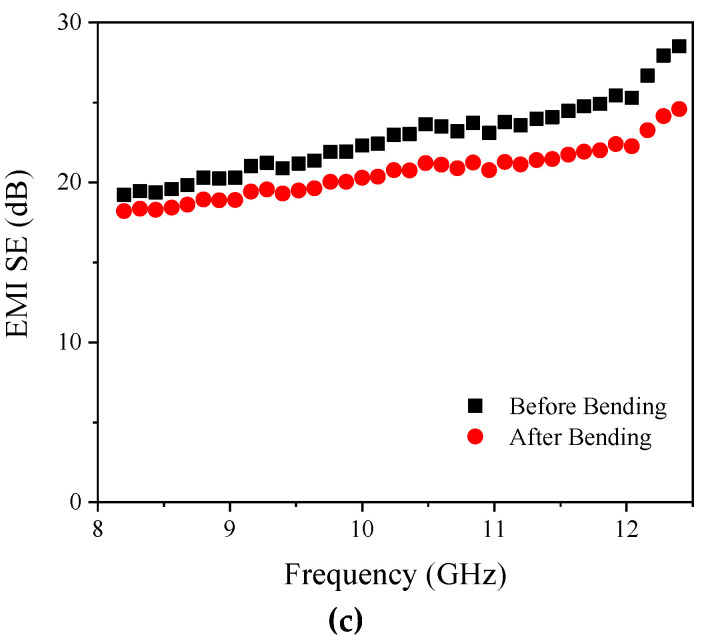
(**a**) and (**b**) SE_T_ and contribution of SE_A_ and SE_R_ of BT/MWCNTs and MWCNTs nanocomposite foams at 10 GHz, respectively; (**c**) EMI SE of the hybrid sample with 10 phr MWCNTs before and after repeated bending.

SE_M_ = 20log(1 − e^−2t/δ^) (7)

where Pi, Pt, σ, f, μ, and δ are the incident and transmitted power, conductivity, frequency, permeability, and skin depth, respectively. Since the shield thickness of the developed foams is greater than skin depth (defined as the distance from the surface of the shield where the EM energy has dropped to 1/e of its initial value at the surface) [42], the effect of multiple reflections can be overlooked [43,44]. The three SE parameters were calculated (Figure 6) for our tested samples with the thickness of 9 mm over the X-band frequencies [41]. The EPDM foamed sample filled with only 20 phr BT was transparent to EM waves and no EMI SE was measured for this sample, due to the low dielectric loss of BT. Meanwhile, the incorporation of MWCNTs in combination with BT into the foams compositions led to the improved shielding efficiencies via both absorption and reflection mechanisms. The presence of free electrons in the structure of MWCNTs results in closed induced electrons current inside the shield by the incident EM wave which enhances the depletion of the wave energy via absorption mechanism [45]. Nevertheless, the foamed EPDM composites composed of BT/MWCNTs exhibited higher values of SE_A_ and SE_R_ than the counterpart samples filled only with MWCNTS or BT. The hybrid EPDM nanocomposite foam loaded with 20 phr BT and 10 phr MWCNTs exhibited a total SE of ~22.3 dB compared to ~16 dB of the 10 phr MWCNTs at 10 GHz (Figure 7a). The improved EM wave absorption and efficiency imparted by BT/MWCNTs hybrid are mainly attributed to the enhanced total polarization as a result of contribution of the space charge polarization, in parallel to the orientational polarization of the dipoles attached to the surfaces of the BT and MWCNTs particles. Moreover, as was discussed, the composite based on 20 phr BT and 10 phr MWCNTs showed higher electrical conductivity than their counterpart (B0C10), which resulted in higher dissipation of the EM wave via conductance loss mechanism. As can be observed in Figure 7a, the SE_A_ shows higher values than SE_R_ over the studied range of frequencies, implying that absorption is the dominant mechanism in the shielding of the EM waves. Although most EMI shielding research is based on polymer matrices, some metal foams have also been studied as a strategy to solve one of the main drawbacks of metals, their weight. Aluminium metal foams, made from melt foaming method [46] and PU sacrifial templates [47], exhibited EMI shielding performance in the X-band of 75 dB and 45 dB, respectively. However, the SE of foams made from melt foaming was not constant throughout the entire frequency range and, hence, part of the EM was transmitted. Additionally, the dominant mechanism in these systems is reflection and they can not be used in applications that require high EM absorbing properties [47,48]. We normalized the total EMI SE values by the density, which is called specific EMI SE (Figure 7b). The specific SE_T_ of hybrid foam containing 10 phr MWCNTs was 25% higher compared with the EPDM/MWCNTs counterpart, in spite of the high density of BT. As the EMI shielding efficiency of materials is governed by various parameters such as conductive particle filler concentration and intrinsic electrical conductivity, type of matrix, thickness, and fabrication process, it is therefore difficult to directly compare the results of different investigations without considering these factors. Hence, we calculated the extent of improvement reported on foamed nanocomposites filled with hybrid fillers as a way to establish a comparative analysis (Table 2). It can be observed that our synthesized EPDM foam filled with BT/MWCNTs (20/10 phr) shows the highest percentage of improvement for SE_T_ (40%), compared to the other polymeric foams reported by others [7,13,34,44,49,50].

For long-life applications of an EM wave shield, high stability of the performance, especially when subjected to repeated bending deformations, is required. Hence, the EMI SE of the prepared EPDM foam composed of BT/MWCNTs (20/10 phr) was measured after 1000 times repeated bending (with a radius of 2.0 mm) (Figure 7c). Interestingly, the EMI SE showed an insignificant decrease over the entire X-band frequency range. The small reduction could be attributed to the high elastic shape recovery resulting from the entropic features of cross-linked EPDM networks assisted by the air molecules trapped within the cells. Only one study has been found reporting a decrease in SE_T_ after repeated bending for polyurethane/reduced graphene oxide nanocomposite foams at 10 GHz [50]. 

**Figure 8:**
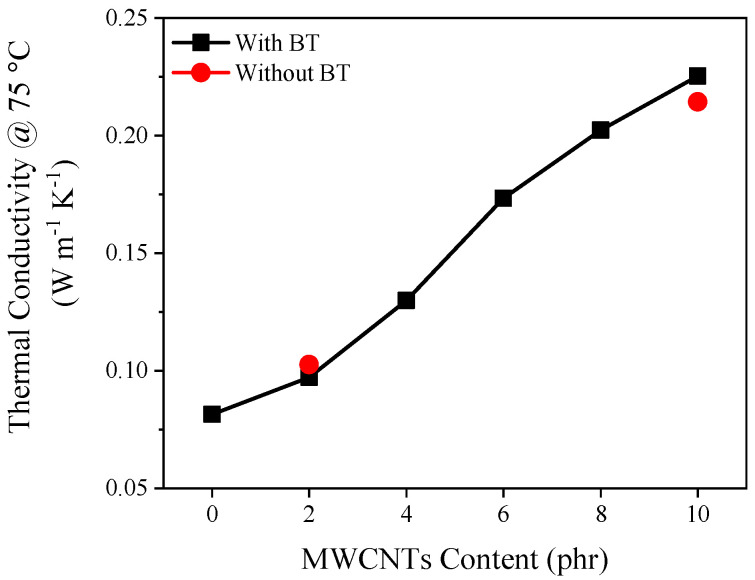
Thermal conductivity of cellular BT/MWCNTs and MWCNTs nanocomposites as a function of MWCNTs loading at 25 °C.

The thermal conductivity of shielding materials is also regarded as an important factor for EMI shielding to minimize the temperature impact on electronic devices. The thermal conductivity of the hybrid EPDM foam showed higher value [0.217 W/(m K)] compared to that measured [0.077 W/(m K)] for the sample composed of only 20 phr BT (Figure 8). This improvement is likely the result of improved thermal conduction through the solid and gas phases of the foam. Both convection and radiation mechanisms can be considered negligible, since the cell size is below 4 mm [51], and the relative density is above 0.2 [52], as reported elsewhere [29]. It is worth mentioning that the thermal conductivity of the MWCNTs foams is similar to their BT/MWCNTs counterparts due to the low volumetric percentage of BT (as the maximum BT volume percentage is about 2 vol.%). Previous studies have shown that a large loading fractions (above 20 vol.%) of BT is needed to obtain a significant increase in the thermal conductivity of polymer composites [53,54].

## 4. Conclusion

In the present study, EMI shielding and absorption have synergistically been improved through the use of a hybrid of conductive and ferroelectric ceramic fillers in the composition of crosslinked EPDM foam. Consequently, while the EPDM foam filled by only 20 phr BT was shown to be transparent to the EM wave within the X-band frequency, the hybrid EPDM nanocomposite foam loaded with 20 phr BT and 10 phr MWCNTs presented a total shielding effectiveness (SE) of ~22.3 dB compared to ~16.0 dB measured for EPDM foam filled with 10 phr MWCNTs. Additionally, the total SE of the BT/MWCNTs foam remained almost constant when subjected to repeated bending due to the high rubber elasticity as well as the elastic memory induced when the sample was deformed. Finally, the BT/MWCNTs-filled EPDM foam showed improved thermal conductivity compared to that of the foamed samples composed of only BT-filled foam. Since other properties, such as resistance to chemicals, moisture, and ozone, are inherent for EPDM rubber, these foams can be regarded as a high potential material for the absorption of high-frequency EM wave in various applications, such as aeronautical, civil, and military.

## Figures and Tables

**Table 1:** Compounds coding and ingredients, in phr (parts per hundred rubber).

Samples	Ingredients								
	EPDM	MA-g-EPDM	MWCNTs	BT	OBSH	Zinc Oxide	Stearic Acid	Dicumyl Peroxide	Sulfur
B20C0	100	5	0	20	5	5	1.5	1	0.5
B20C2	100	5	2	20	5	5	1.5	1	0.5
B20C4	100	5	4	20	5	5	1.5	1	0.5
B20C6	100	5	6	20	5	5	1.5	1	0.5
B20C8	100	5	8	20	5	5	1.5	1	0.5
B20C10	100	5	10	20	5	5	1.5	1	0.5
B0C2	100	5	2	0	5	5	1.5	1	0.5
B0C10	100	5	10	0	5	5	1.5	1	0.5

**Table 2:** Comparison of the improvement percentages of the EMI SE in hybrid nanocomposite foams in the X-band.

Materials	SE_T_ (%)	SE_A_ (%)	SE_R_ (%)	Fillers	Ref.
PEI	38	43	67	Graphene/Fe3O4	[13,34]
Silicone rubber	18	22	−85	MWCNTs/Fe3O4	[44]
Epoxy	34.5	41	−56	Acetylene black/nickel coated carbon fiber	[7]
Epoxy	33.5	-	-	MWCNTs/nickel-plated carbon fibers	[49]
Epoxy	30	-	-	MWCNTs/Fe3O4@Ag	[50]
EPDM	40	42	18	BT/MWCNTs	This work
